# Regulatory Roles of Phospholipase A_2_ Enzymes and Bioactive Lipids in Mast Cell Biology

**DOI:** 10.3389/fimmu.2022.923265

**Published:** 2022-06-27

**Authors:** Yoshitaka Taketomi, Makoto Murakami

**Affiliations:** Laboratory of Microenvironmental and Metabolic Health Sciences, Center for Disease Biology and Integrative Medicine, Graduate School of Medicine, The University of Tokyo, Bunkyo-ku, Japan

**Keywords:** phospholipid, phospholipase A_2_, lipid mediator, mast cells, allergy, type 2 immunity

## Abstract

Lipids play fundamental roles in life as an essential component of cell membranes, as a major source of energy, as a body surface barrier, and as signaling molecules that transmit intracellular and intercellular signals. Lipid mediators, a group of bioactive lipids that mediates intercellular signals, are produced *via* specific biosynthetic enzymes and transmit signals *via* specific receptors. Mast cells, a tissue-resident immune cell population, produce several lipid mediators that contribute to exacerbation or amelioration of allergic responses and also non-allergic inflammation, host defense, cancer and fibrosis by controlling the functions of microenvironmental cells as well as mast cell themselves in paracrine and autocrine fashions. Additionally, several bioactive lipids produced by stromal cells regulate the differentiation, maturation and activation of neighboring mast cells. Many of the bioactive lipids are stored in membrane phospholipids as precursor forms and released spatiotemporally by phospholipase A_2_ (PLA_2_) enzymes. Through a series of studies employing gene targeting and lipidomics, several enzymes belonging to the PLA_2_ superfamily have been demonstrated to participate in mast cell-related diseases by mobilizing unique bioactive lipids in multiple ways. In this review, we provide an overview of our current understanding of the regulatory roles of several PLA_2_-driven lipid pathways in mast cell biology.

## Introduction

Altered tissue sensitivity to environmental triggers contributes to the development of allergic inflammation, which is often associated with type 2 immunity (1, 2). Allergic diseases have become very common in hygienic countries, with a prevalence that has increased by 2–3-fold within the last two decades. Patients with anaphylaxis, food allergy, asthma, allergic rhinitis, and atopic eczema typically have elevated levels of serum IgE in association with activation of mast cells (3). Development of mast cells in extravascular tissues depends essentially on the stromal cytokine stem cell factor (SCF) and its receptor c-Kit, with various cytokines and adhesion molecules having accessory roles in tissue- or disease-specific contexts (4). Crosslinking of FcϵRI, a high affinity IgE receptor on the surface of mast cells, with IgE and antigen (allergen), or exposure to several IgE-independent stimuli such as substance P, which is released from TRPV1^+^ sensory neurons and acts on the MRGPR family receptors on mast cells (5), triggers exocytosis of granule contents (*e.g.*, histamine and proteases) and production of various cytokines and chemokines, thereby promoting harmful allergic reactions and also participating in beneficial host defense against invading microorganisms or venom components (6). Mast cells also generate a variety of bioactive lipids called lipid mediators, which take part in fine-tuning of allergic responses by regulating the functions of various cell types. Furthermore, proper maturation and activation of mast cells are positively or negatively affected by various microenvironmental factors, including bioactive or structural lipids. For instance, the CD300 immunoreceptor family binds to structural lipids (*e.g.*, ceramides, sphingomyelin, phosphatidylserine (PS) and phosphatidylethanolamine (PE)) as functional ligands and negatively regulates FcϵRI signaling to put a brake on excessive immediate allergic reactions (7, 8).

Lipid mediators, on which we put a specific focus in this article, are produced through specific biosynthetic pathways, are released extracellularly *via* diffusion, specific transporters or extracellular vesicles (EVs), act on specific receptors on target cells, and are rapidly degraded or inactivated within local tissue microenvironments. Individual lipid mediators display pleiotropic functions and can have both offensive and protective effects by acting on distinct receptor subtypes expressed on different cell types. Lipid mediators are categorized into several classes, including eicosanoids derived from ω6 arachidonic acid (AA), such as prostaglandins (PGs) and leukotrienes (LTs); specialized pro-resolving lipid mediators (SPMs) derived from ω3 eicosapentaenoic acid (EPA) or docosahexaenoic acid (DHA), such as resolvins, maresins and protectins; and lysophospholipid-derived mediators, such as lysophosphatidic acid (LPA) and platelet-activating factor (PAF) (9–12). Biosynthesis of these lipid mediators is initiated in many if not all cases by hydrolysis of membrane phospholipids by phospholipase A_2_s (PLA_2_s). The mammalian genome encodes more than 50 PLA_2_-related enzymes, which are classified into several structurally related families, including the cytosolic PLA_2_ (cPLA_2_), Ca^2+^-independent PLA_2_ (iPLA_2_), secreted PLA_2_ (sPLA_2_), PAF acetylhydrolase (PAF-AH), lysosomal PLA_2_, PLA-acyltransferase (PLAAT), and α/β hydrolase (ABHD) families (13). The properties and functions of individual PLA_2_s, as revealed by studies using knockout or transgenic mice for individual PLA_2_s in combination with comprehensive lipidomics, have been summarized in current reviews (14, 15).

Importantly, genetic and pharmacological studies have provided evidence that several intracellular and extracellular PLA_2_s uniquely regulate the maturation and functions of mast cells through driving distinct lipid pathways *in vivo* (16–23). These include (i) cPLA_2_α-driven generation of AA metabolites by activated mast cells for propagation or sequestration of allergic inflammation, (ii) PAF-AH2-driven constitutive generation of EPA/DHA metabolites for optimization of mast cell activation, (iii) sPLA_2_-III-driven paracrine PGD_2_ circuit for mast cell maturation, (iv) sPLA_2_-IIA-driven modulation of gut microbiota that indirectly affects mast cells, and (v) miscellaneous PLA_2_s whose roles in mast cells are controversial. In this article, we will make an overview of the roles of these PLA_2_-driven lipid pathways in mast cell biology.

## Regulatory Roles of Intracellular PLA_2_s in Mast Cell Biology

### Eicosanoid Generation by cPLA_2_α in Activated Mast Cells

**Figure 1:**
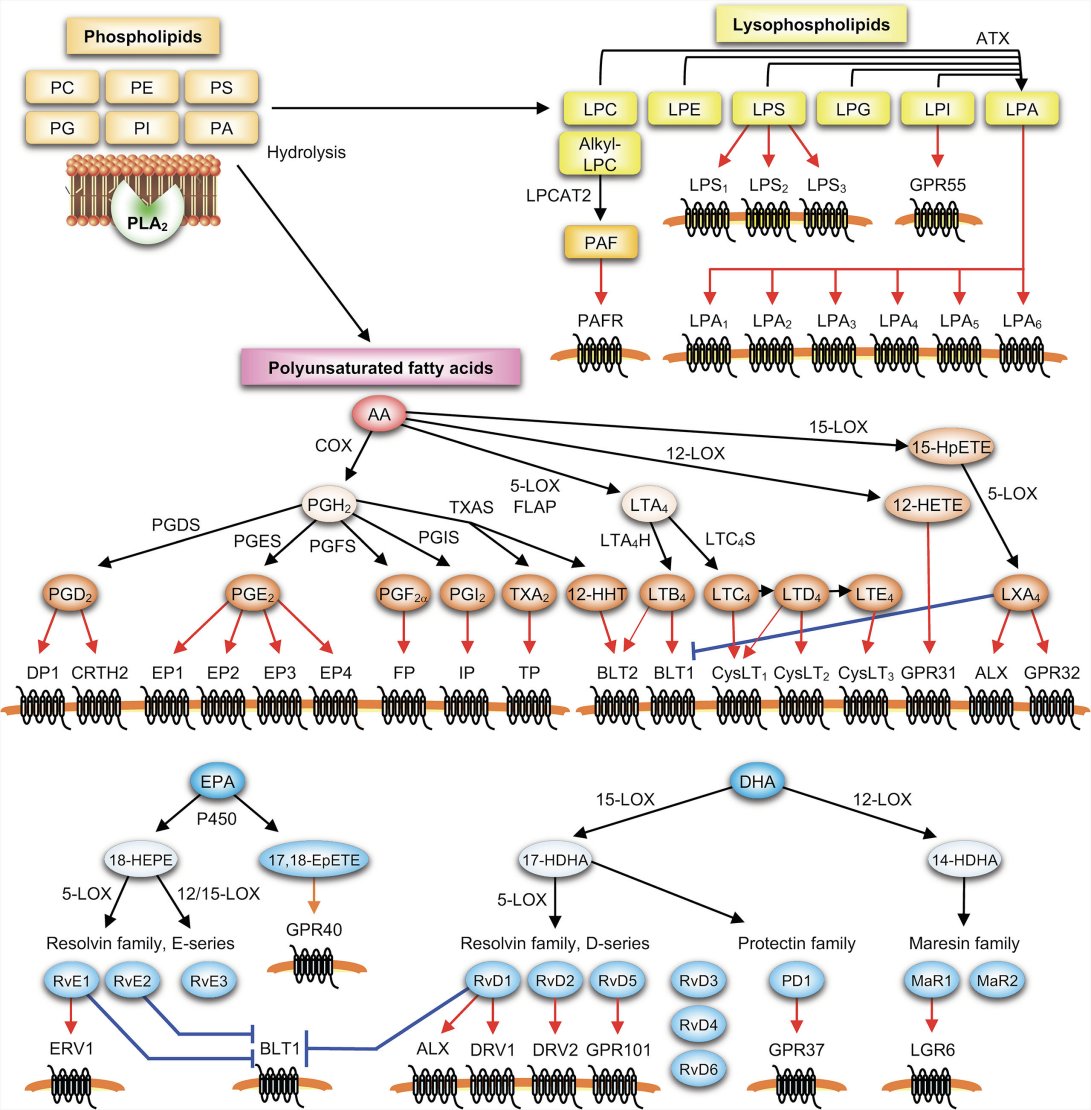
PLA_2_-driven lipid mediator pathways. Lysophospholipids (such as lysophosphatidylcholine (LPC), lysophosphatidylethanolamine (LPE), lysophosphatidylserine (LPS), lysophosphatidylglycerol (LPG), lysophosphatidylinositol (LPI) and LPA) and polyunsaturated fatty acids (such as AA, EPA and DHA) released from membrane phospholipids (PC, PE, PS, phosphatidylinositol (PI), phosphatidylglycerol (PG) and phosphatidic acid (PA)) by PLA_2_ are metabolized by downstream enzymes into various lipid mediators, which in turn act on their specific receptors on target cells. In the PLA_2_-lysophospholipid axis, various lysophospholipids are converted by autotaxin (ATX) into LPA, and alkyl-LPC is converted by LPC acyltransferase 2 (LPCAT2) into PAF. In the PLA_2_-PUFA axis, ω6 AA released by PLA_2_ is metabolized into prostanoids including PGD_2_, PGE_2_, PGF_2α_, PGI_2_, TXA_2_, and 12-hydroxyheptadecatrenoic acid (12-HHT) *via* the COX pathway involving COX-1 or COX-2 and terminal PG synthases (PGDS, PGES, PGF_2α_ synthase (PGFS), PGI_2_ synthase (PGIS) and TXA_2_ synthase (TXAS)), or into LTB_4_ and cysLTs (LTC_4_, D_4_ and E_4_) *via* the 5-LOX pathway involving 5-LOX, its cofactor 5-LOX-activating protein (FLAP), and terminal LT synthases (LTA_4_H and LTC_4_S). Combined actions of 15-LOX and 5-LOX give rise to lipoxins (*e.g*., LXA_4_), an AA-derived SPM. ω3 PUFAs (*e.g*., EPA and DHA) are metabolized by LOXs and/or CYP450s into various SPMs including hydroxy-EPA (*e.g*., 18-HEPE), hydroxy-DHA (*e.g*., 14- and 17-HDHAs), E- and D-series resolvins (*e.g*., RvE1-3 and RvD1-6), protectin D1 (PD1), maresins (*e.g*., MaR1-2), and ω3 epoxides (e.g., 17,18-EpETE).

In general, PLA_2_ acts as the most upstream enzyme in the biosynthetic pathways for various lipid mediators derived from membrane phospholipids (**Figure 1**). It is well established that group IVA cPLA_2_ (cPLA_2_α or PLA2G4A), the only PLA_2_ subtype that shows a striking substrate specificity for AA-containing phospholipids, is essential for stimulus-coupled release of AA from phospholipids and thereby production of eicosanoids in many cell types including mast cells (24, 25). cPLA_2_α has an *N*-terminal C2 domain, which allows translocation of the enzyme from the cytosol to perinuclear, ER and Golgi membranes in response to an increase in cytosolic Ca^2+^ following cell activation (24). In addition, cPLA_2_α is phosphorylated by mitogen-activated protein kinases (MAPKs) and possibly other kinases, an event that is essential for full activation of cPLA_2_α (25). Phosphatidylinositol-4,5-bisphosphate (PIP_2_) and ceramide-1-phosphate (C1P) modulate the subcellular localization and activation of cPLA_2_α (26, 27). These properties place cPLA_2_α as a central regulator of the stimulus-coupled generation of eicosanoids, a class of AA-derived lipid mediators including PGs and LTs.

**Figure 2:**
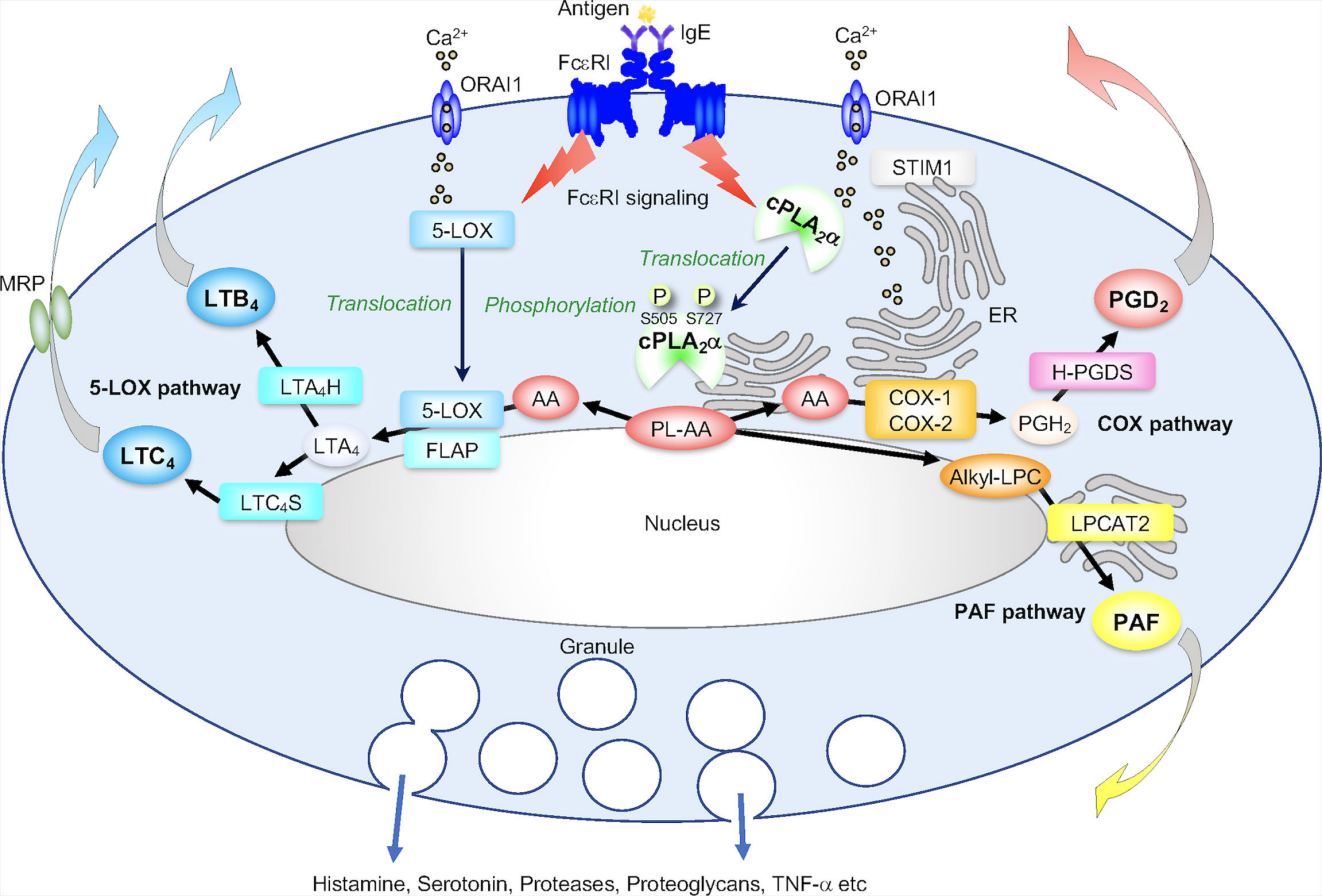
The cPLA_2_α-eicosanoid axis. (A) The role of cPLA_2_α in generation of PGD_2_ and LTs in IgE/antigen (Ag)-activated mast cells. Following FcεRI-dependent signaling that is linked to STIM1/ORAI1-driven Ca^2+^ influx, cPLA_2_α translocates from the cytosol to the perinuclear, Golgi and ER membranes, where several downstream eicosanoid-biosynthetic enzymes are also located. The activity of cPLA_2_α is augmented by phosphorylation at Ser^505^ by MAPKs, Ser^727^ by MAPK-activated protein kinases (MAPKAPKs), and possibly other kinases. The AA released from AA-containing phospholipids (PL-AA) by cPLA_2_α is then metabolized into PGD_2_ *via* the COX pathway involving COX-1 (or COX-2 if it is induced) and H-PGDS and into LTB_4_ and LTC_4_ *via* the 5-LOX pathway involving 5-LOX (which also translocates from the cytosol to the perinuclear membrane in response to Ca^2+^), FLAP (a cofactor that presents AA to 5-LOX), LTA_4_H and LTC_4_S. Alternatively, cPLA_2_α-generated alkyl-LPC is acetylated by LPCAT2 to PAF. These reactions occur independently of degranulation and cytokine induction.

Activated mast cells produce LTB_4_, LTC_4_ and PGD_2_ as major AA-derived eicosanoids. Following FcεRI-dependent or -independent activation of mast cells, the AA released by cPLA_2_α from membrane phosphatidylcholine (PC) and phosphatidylethanolamine (PE) is converted by the sequential action of 5-lipoxygenase (LOX) and terminal LT synthases to LTs (the 5-LOX pathway), or by that of cyclooxygenases (COXs) and hematopoietic PGD synthase (H-PGDS) to PGD_2_ (the COX pathway) (**Figure 2**), with preferential production of PGD_2_ by connective-tissue mast cells (CTMCs) and LTC_4_ by mucosal mast cells (MMCs) (28–30). After being exported from mast cells *via* the specific transporter MRP1, LTC_4_ is converted to LTD_4_ and then to LTE_4_ by extracellular peptidases; therefore, these three LTs, which have a glutathione-derived cysteine in their structures, are often referred to as cysteinyl LTs (cysLTs) (31). Of the two COX isoforms COX-1 and -2, pre-existing COX-1 is mainly responsible for immediate PGD_2_ generation that occurs within a few minutes, while inducible COX-2 is responsible for delayed PGD_2_ generation that lasts for several hours (32, 33). IL-3-driven bone marrow-derived mast cells (BMMCs), a relatively immature mast cell population, produce LTC_4_ in preference to PGD_2_, thus resembling MMCs. Coculture of BMMCs with fibroblasts in the presence of SCF facilitates their maturation toward a CTMC-like phenotype, with an eicosanoid balance shift from LTs to PGD_2_ that is accompanied by increased expression of cPLA_2_α, COX-2, H-PGDS and LTB_4_ dehydrogenase (which inactivates LTB_4_) and by decreased expression of LTA_4_ hydrolase (LTA_4_H; LTB_4_ synthase) and LTC_4_ synthase (LTC_4_S) (34, 35).

Following FcεRI-dependent or -independent activation, BMMCs from cPLA_2_α-deficient (*Pla2g4a* ^–/–^) mice fail to produce LTs and PGD_2_ as well as PAF, a lysophospholipid-derived lipid mediator that also participates in allergic responses, with no change (17) or a slight increase (36, 37) in degranulation. *Pla2g4a* ^–/–^ mice or WT mice treated with a cPLA_2_α inhibitor display reduced asthmatic responses upon pulmonary antigen challenge (16, 38). As in mice lacking cPLA_2_α, asthmatic responses are attenuated in mice lacking biosynthetic enzymes or receptors for LTs, PGD_2_, or PAF (39–45), implying a critical role of the cPLA_2_α-LT/PGD_2_/PAF axis in this pulmonary allergic disease. In contrast, passive cutaneous anaphylaxis (PCA), a model of immediate-type allergic reactions, is not altered in *Pla2g4a* ^–/–^ mice, likely due to simultaneous shutdown of the generation of pro-anaphylactic LTC_4_ and anti-anaphylactic PGD_2_ by mast cells (20).

LTB_4_ is a potent chemoattractant for leukocytes, while cysLTs promote microvascular permeability, sustained smooth muscle contraction, and mucus secretion. Among the three cysLT receptors (CysLT_1~3_), CysLT_1_ binds to LTD_4_ > LTC_4_ > LTE_4_, CysLT_2_ to LTC_4_ = LTD_4_ > LTE_4_, and CysLT_3_ to LTE_4_ as determined by transfection systems, although their specificity *in vivo* is less clear. Besides being produced by mast cells as well as by macrophages, eosinophils and basophils, cysLTs can also activate mast cells to produce contractile PGs *via* a mechanism involving a receptor sensitive to montelukast (46, 47), a CysLT_1_ antagonist that is now clinically used for treating patients with asthma (48). IL-4-dependent mast cell proliferation requires autocrine/paracrine cysLT signaling *via* CysLT_1_, and CysLT_2_ interacts with CysLT_1_ to dampen cysLT-dependent mitogenic responses of mast cells (49, 50). Recently, cysLTs have attracted attention as critical regulators of group 2 innate lymphoid cells (ILC2s). CysLTs induce CysLT_1_-dependent production of Th2 cytokines (IL-4, IL-5, and IL-13) by ILC2s in cooperation with the epithelial-derived cytokines IL-25 and IL-33, promoting the expansion of ILC2s, Th2 cells, and eosinophils after allergen challenge (51–53). CysLT_2_-driven production of IL-33 by type 2 alveolar cells leads to expansion of ILC2s, and pharmacological inhibition of CysLT_2_ blocks IL-33-driven mast cell activation and associated lung inflammation (54). Antigen inhalation leads to expansion of IL-25-producing airway brush cells or tuft cells, which is attenuated by genetic deletion of LTC_4_S or CysLT_3_, revealing an importance of the airway LTE_4_-CysLT_3_ axis in type 2 inflammation (55). Similarly, cysLTs produced by intestinal tuft cells cooperate with IL-25 to activate ILC2s, and tuft cell-specific deletion of cysLT generation blocks type 2 immune responses against food allergens or helminth infection (56). In the context of atopic dermatitis, tape stripping of the stratus corneum (equivalent to scratching) causes keratinocytes to systemically release IL-33, which synergizes with intestinal tuft cell-derived IL-25 and cysLTs to drive the expansion and activation of ILC2s that produce IL-4, eventually promoting mast cell expansion in the intestine (57). In addition, LTC_4_ derived from basophils rather than mast cells acts on CysLT_2_ on natriuretic polypeptide-positive (Nppb^+^) sensory neurons to elicit itch (58, 59), underscoring a novel mechanism for acute itch flares in atopic dermatitis.

PGD_2_ exerts pro- and anti-allergic functions through two PGD receptors, DP1 and CRTH2 (DP2), depending on the disease contexts. Global or mast cell-specific deletion of DP1 ameliorates asthma and anaphylaxis, which may be attributable in part to the impaired maturation of mast cells (see below) (20, 43). In contrast, DP1 deficiency increases the migration of antigen-captured dendritic cells into the draining lymph nodes, thereby exacerbating contact hypersensitivity (CHS), a Th1-dependent delayed-type allergic response (60, 61). Global or mast cell-specific deletion of H-PGDS worsens anaphylaxis (20, 62) and food allergy with mast cell hyperplasia (63). The anti-inflammatory action of PGD_2_ is mediated, at least in part, by its non-enzymatic conversion to 15-deoxy-PGJ_2_, which acts on the nuclear receptor PPARγ to attenuate pro-inflammatory NF-κB signaling (64). Mast cell-derived PGD_2_ is involved in ILC2 expansion *via* CRTH2 (65), and CRTH2-deficient mice exhibit reduced pulmonary ILC2 responses and type 2 inflammation, an event that is rescued by transfer of CRTH2-sufficient ILC2s (66). LTE_4_ enhances the ability of PGD_2_ to induce ILC2 and Th2 cells (52, 67), implying a synergistic role of the two mast cell-derived eicosanoids in promoting type 2 immunity. Activation of cPLA_2_α in intestinal tuft cells leads to generation of not only LTC_4_ (see above) but also PGD_2_, the latter of which increases mucus secretion by goblet cells to impede pathogen invasion (68). Thus, it seems that mast cells and tuft cells share common characteristics in that they produce both PGD_2_ (via the cPLA_2_α/COX-2/H-PGDS pathway) and LTC_4_ (via the cPLA_2_α/5-LOX/LTC_4_S pathway) in response to specific stimuli and participate in fine-tuning allergic responses as well as host defense against pathogens.

**Figure 3:**
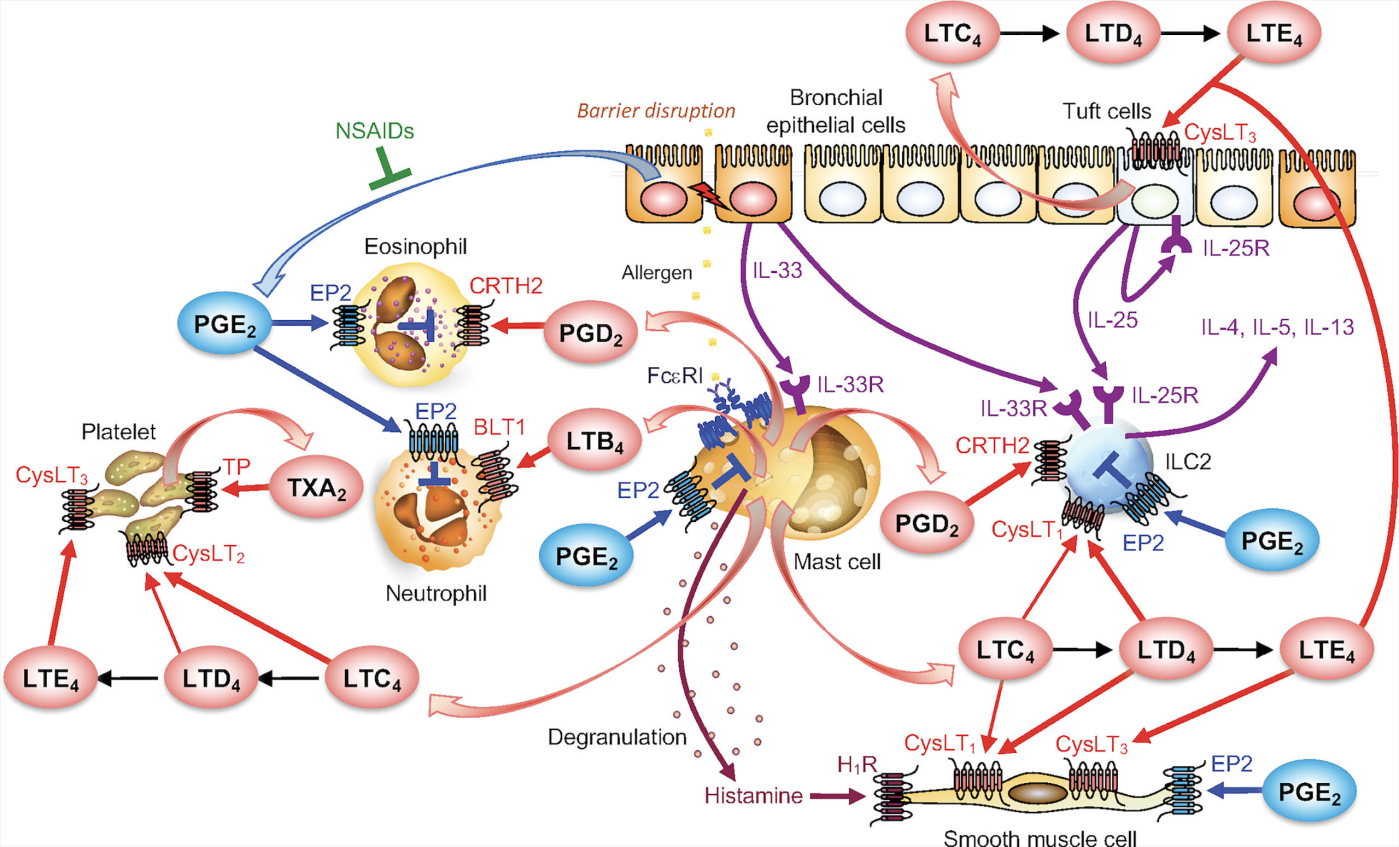
The mast cell-driven eicosanoid network in asthma. PGD_2_ and LTs produced by activated mast cells act on various cell types including leukocytes, platelets, bronchial epithelial cells and smooth muscle cells to promote allergic responses. PGE_2_, produced by various cells such as bronchial epithelial cells (but not mast cells), dampens allergic responses. NSAIDs (COX inhibitors) blocks PGE_2_ production, thereby exacerbates asthma known as AERD. For details, please see the text.

Although mast cells produce PGE_2_ minimally, this pleiotropic eicosanoid is produced by stromal cells surrounding mast cells and plays an anti-allergic role in general by acting on four types of its receptor (EP1–4). During mast cell-fibroblast coculture, the AA released by cPLA_2_α in mast cells is transferred to adjacent fibroblasts through the transcellular route and then metabolized there by PGE_2_ synthase (mPGES-1) to PGE_2_ (17), which acts on stromal EP3 to counteract allergic reaction. As such, mice lacking EP3 display more severe asthma, anaphylaxis, and CHS (20, 69, 70). Paradoxically, however, PGE_2_ directly elicits mast cell activation *via* EP3 by evoking Ca^2+^ signaling (71). Genetic deletion of mPGES-1 or pharmacological inhibition of COX-1 rather than COX-2 mimics aspirin-exacerbated respiratory disease (AERD) that is characterized by bronchoconstriction, eosinophilia, and mucus secretion upon exposure to non-steroidal anti-inflammatory drugs (NSAIDs; COX inhibitors), an event that is reversed by EP2 (or to a lesser extent EP1) agonist (72). In this AERD pathology, aspirin-induced mast cell activation and cysLT overproduction depend on the adherent interaction of platelets with granulocytes and thromboxane A_2_ (TXA_2_) signaling (72, 73). In addition, PGE_2_ alleviates mast cell activation (74–76), increases vascular remodeling (77), prevents eosinophilia (78, 79), reduces proliferation of ILC2s and Th2 cells (80, 81), and attenuates atopic dermatitis by reducing TSLP expression (82) *via* EP2 signaling. The mast cell-related eicosanoid network is illustrated in **Figure 3**.

### Constitutive Generation of ω3 Epoxides by PAF-AH2 in Mast Cells

**Figure 4:**
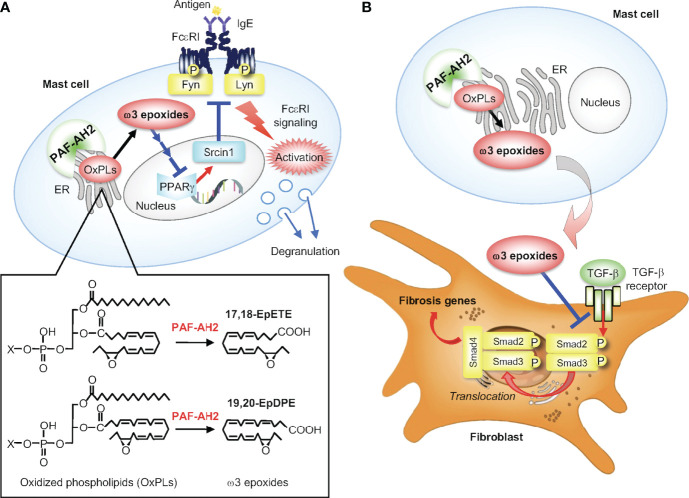
The PAF-AH2-ω3 epoxide axis. **(A)** PAF-AH2-driven constitutive generation of ω3 epoxides in mast cells and their cell-autonomous role in augmentation of FcεRI signaling. PAF-AH2 directly hydrolyzes phospholipids with oxidized fatty acids (ω3 epoxides in this case) to release free ω3 epoxides (17,18-EpETE and 19,20-EpDPE), which act on the nuclear receptor PPARγ (probably indirectly) to suppress the expression of Srcin1, a Src-inhibitory protein. This Srcin1 downregulation eventually leads to increased activation of the FcεRI-proximal Src family kinases Fyn and Lyn, thereby ensuring optimal FcεRI signaling. **(B)** Suppression of pulmonary hypertension by PAF-AH2-derived ω3 epoxides. The ω3 epoxides released from lung mast cells acts on neighboring fibroblasts in a paracrine manner to prevent TGF-β-driven Smad2 phosphorylation, thereby attenuating perivascular fibrosis leading to amelioration of pulmonary hypertension.

PAF-AHs were originally identified as a unique group of PLA_2_s having the capacity to hydrolyze and thereby inactivate PAF (83, 84). Plasma-type PAF-AH (PLA2G7 or group VIIA PLA_2_) is a secreted protein produced by macrophages, mast cells or other sources, and is now more generally referred to as lipoprotein-associated PLA_2_ (Lp-PLA_2_), existing as a low-density lipoprotein (LDL)-bound form in human plasma (85). PAF-AH2 (group VIIB PLA_2_) is a cytosolic enzyme that shows significant structural homology with Lp-PLA_2_. Despite their names, however, there has been no confirmative evidence that Lp-PLA_2_ or PAF-AH2 has a protective role against allergic reactions by degrading PAF *in vivo*. Rather, it is now recognized that Lp-PLA_2_ and PAF-AH2 preferentially hydrolyze oxidized phospholipids in lipoproteins and cells, respectively. Importantly, PAF-AH2 is abundantly expressed in mast cells and contributes to constitutive (*i.e.*, stimulus-independent) generation of unique epoxy-metabolites of ω3 fatty acids (ω3 epoxides), which prime mast cells to ensure optimal FcεRI-dependent activation (**Figure 4A**) and attenuates pulmonary hypertension by reducing the expression of pro-fibrotic factors (**Figure 4B**) in autocrine and paracrine manners, respectively (18, 19).

Comprehensive lipidomics profiling of BMMCs revealed that unique ω3 epoxides, namely 17,18-epoxyeicosatetraenoic acid (17,18-EpETE) and 19,20-epoxydocosapentaenoic acid (19,20-EpDPE) derived respectively from EPA and DHA, are constitutively released from the cells (18). Unlike the canonical route for eicosanoid biosynthesis, in which the AA released by cPLA_2_α from membrane phospholipids is metabolized by downstream enzymes to eicosanoids (see above), these ω3 epoxides are liberated directly by PAF-AH2 from ω3 epoxide-esterified phospholipids pre-existing in cell membranes (**Figure 4A**). Genetic or pharmacological inactivation of PAF-AH2 shuts off the constitutive release of ω3 epoxides from mast cell membranes, resulting in reduced FcεRI signaling and anaphylaxis. Supplementation of *Pafah2* ^–/–^ BMMCs with ω3 epoxides fully restores the defective FcεRI-dependent, but not -independent, activation. Mechanistically, ω3 epoxides augment FcεRI-driven activation of mast cells by downregulating Srcin1, a Src-inhibitory protein that counteracts the FcεRI-proximal Src-family tyrosine kinases Fyn and Lyn and thereby dampens FcϵRI signaling (18). Transcriptome analysis revealed that genes regulated by ω3 epoxides largely overlap with those regulated by a PPARγ antagonist in *Pafah2* ^–/–^ BMMCs, implying that the action of ω3 epoxides may involve the inactivation of PPARγ, a lipid-sensing nuclear receptor that induces Srcin1 expression (18).

In general, ω3 fatty acids including EPA and DHA play beneficial roles in various biological events. These ω3 fatty acids act by themselves on the fatty acid receptors GPR40 or GPR120 (86, 87), or are converted by LOX or cytochrome P450 (CYP450) enzymes to SPMs with potent anti-inflammatory or pro-resolving functions. Indeed, several CYP450 isoforms have the capacity to catalyze the epoxidation of EPA and DHA to yield ω3 epoxides (88). Individual SPMs act on their specific receptors, such as ERV1/ChemR23 for resolvin E1 (RvE1), DRV1/GPR32 for RvD1, DRV2/GPR18 for RvD2, GPR37 for protectin D1 (PD1), and LGR6 for maresin 1 (MaR1) (89–93), and can limit leukocyte recruitment, induce granulocyte apoptosis, enhance efferocytosis by phagocytes, facilitate the switch from M1 to M2 macrophages, promote the return of non-apoptotic cells to lymphatics and blood vessels, and help tissue repair (11, 94, 95). In this context, it seems strange that ω3 epoxides potentiate (rather than attenuate) mast cell activation and thereby exacerbate allergic responses. It should be noted, however, that the immunosuppressive action of SPMs can be disadvantageous to host defense (11, 94, 95). Conceivably, FcϵRI-induced mast cell activation might have evolved originally as a defense system against harmful venom, bacteria, and/or parasites (96, 97), where the ω3 epoxide-mediated optimization of FcϵRI signaling may be important for proper elimination of these unfavorable materials from the body and thereby maintenance of a healthy state. In a modern hygienic environment, however, this protective response has been shifting into deleterious outcomes against exposure to environmental allergens, manifesting as allergic diseases.

In contrast to their augmentative role in FcεRI-dependent mast cell activation and anaphylaxis, ω3 epoxides produced by PAF-AH2 in mast cells have a protective role in pulmonary hypertension, a fatal rare disease that causes right heart failure by elevated pulmonary arterial resistance (19). Global or mast cell-specific deletion of PAF-AH2 in mice accelerates vascular remodeling with perivascular fibrosis, resulting in exacerbation of hypoxic pulmonary hypertension. Mechanistically, 17,18-EpETE and 19,20-EpDPE produced by PAF-AH2 in lung mast cells act on stromal fibroblasts in a paracrine manner to suppress their activation by inhibiting TGF-β-driven Smad2 signaling (**Figure 4B**). Administration of ω3 epoxides into mice improves pulmonary hypertension by reducing advanced vascular remodeling harboring perivascular fibrosis in several models. Furthermore, the whole-exome sequencing of patients with pulmonary arterial hypertension identifies two candidate pathogenic variants of the *PAFAH2* gene. Thus, the PAF-AH2–ω3 epoxide axis could be a promising therapeutic target for pulmonary hypertension. In addition, 17,18-EpETE suppresses CHS by inhibiting neutrophil migration through GPR40 (98) Thus, the actions of ω3 epoxides are context-dependent, having detrimental or beneficial effects depending on the diseases.

Plasma-type PAF-AH (PLA2G7/Lp-PLA_2_) is abundantly expressed in BMMCs, is secreted after FcεRI-dependent activation, and may participate in the degradation of PAF produced by these cells in an autocrine fashion (99). Interestingly, a comprehensive transcriptome analysis of various mast cell populations demonstrated that the expression level of the *Pla2g7* gene in skin mast cells is much lower than that in tongue, tracheal, esophageal and peritoneal mast cells (100). Although the physiological significance of the low expression of this enzyme in skin mast cells is unclear, it might avoid rapid degradation of PAF produced by these cells or neighboring cells in a local skin niche. In the aforementioned pulmonary hypertension model, *Pla2g7* ^–/–^ mice do not display any noticeable phenotype (19), implying the segregated role of the two (plasma-type and intracellular) PAF-AH isoforms in this disease. 

## Regulatory Roles of Extracellular PLA_2_s in Mast Cell Biology

### General View of sPLA_2_s

The sPLA_2_ family comprises Ca^2+^-dependent, low-molecular-mass enzymes with a conserved His-Asp catalytic dyad. There are 11 mammalian sPLA_2_s (catalytically active IB, IIA, IIC, IID, IIE, IIF, III, V, X and XIIA and inactive XIIB), which are structurally subdivided into group I/II/V/X, group III, and group XII branches (101). Individual sPLA_2_s exhibit distinct tissue distributions and exert their specific functions in lipid mediator-dependent or -independent fashions (102–105). In extracellular milieus, sPLA_2_s act on the plasma membrane of activated, damaged, or dying cells (rather than that of resting cells) and also on non-cellular phospholipid components, such as dietary food, lipoproteins, lung surfactant, EVs, and membranes of invading microbes, as hydrolytic targets. Although the expression and potential functions of sPLA_2_s in mast cells had been reported in previous studies (106–113), the results should be interpreted with caution since many of them relied on the strategies employing overexpression or exogenous addition of super-physiologic levels of sPLA_2_s, which might not precisely reflect pathophysiologic situations *in vivo*. Beyond this caveat, gene targeting studies have provided unequivocal evidence for the involvement of several sPLA_2_s in the regulation of mast cells in cell-autonomous and non-autonomous ways. Our comprehensive phenotypic screening of various sPLA_2_ knockout strains, with mast cell-dependent PCA reaction *in vivo* and BMMC functions *ex vivo* as readouts, has revealed that mice null for sPLA_2_-III and -IIA, but not those for sPLA_2_-IB, -IID, -IIE, -IIF, -V and -X, displayed notable alterations in the maturation and/or functions of mast cells (20), as described below.

### Regulation of Mast Cell Maturation by sPLA_2_-III Through the Paracrine PGD_2_ Pathway

**Figure 5:**
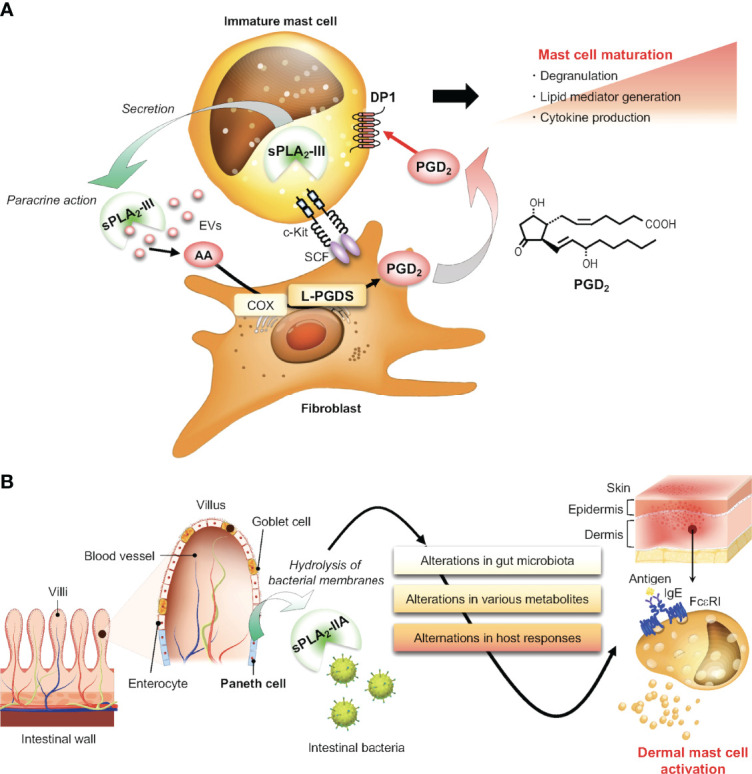
Regulatory roles of sPLA_2_s in mast cell biology. **(A)** The sPLA_2_-III-driven paracrine PGD_2_ circuit for proper mast cell maturation. sPLA_2_-III secreted from immature mast cells hydrolyzes phospholipids in adjacent fibroblast membranes or possibly in mast cell-derived EVs to release AA. This AA is metabolized by fibroblastic COX/L-PGDS to PGD_2_, which in turn acts on DP1 on mast cells to promote mast cell maturation. **(B)** Non-canonical action of sPLA_2_-IIA on mast cells *via* shaping of the gut microbiota. sPLA_2_-IIA, a potent bactericidal protein, is secreted from intestinal Paneth cells and modulates the gut microbiota. This event has systemic effects on immunity and metabolism, thereby secondarily affecting mast cell activation in distal tissues.

Bee venom group III PLA_2_ (bvPLA_2_), when injected into mouse skin, directly activates mast cells likely through hydrolysis of membrane phospholipids to give rise to lysophospholipids, whose massive accumulation can cause lysis of all the cells (including mast cells) in the milieu due to their detergent-like nature leading to release of the alarmin IL-33 and thereby activation of ILC2s and then Th2 cells (114). The aggravated Th2 response by bvPLA_2_ can be considered as a protective mechanism against future exposure to this noxious venom component. Administration of human sPLA_2_-III, the sole mammalian homolog of bvPLA_2_, into mouse skin also elicits mast cell activation (20). Endogenous sPLA_2_-III is stored in and released from secretory granules of mouse and human mast cells. Importantly, mast cell-dependent passive and active anaphylactic responses are markedly reduced in mice lacking sPLA_2_-III (*Pla2g3* ^–/–^) and conversely augmented in mice with transgenic overexpression of human sPLA_2_-III (*PLA2G3^TG^*) (20). Notably, mast cells in *Pla2g3* ^–/–^ mice are numerically normal but morphologically and functionally immature, indicating that sPLA_2_-III does not merely act as a mast cell activator, but also facilitates mast cell maturation. In fact, histamine and protease contents in secretory granules, expression of mast cell maturation markers (*e.g*., histidine decarboxylase (histamine synthase), mast cell proteases, and H-PGDS), and FcεRI-dependent and even -independent activation are considerably lower in *Pla2g3* ^–/–^ mast cells than in *Pla2g3* ^+/+^ mast cells. These phenotypes are mast cell-autonomous, since BMMCs from *Pla2g3* ^–/–^ mice fail to reconstitute the anaphylactic response after their transfer into mast cell-deficient *Kit* ^W-sh/W-sh^ mice, and since mast cell-specific deletion of sPLA_2_-III also leads to similar defects in mast cell maturation and anaphylaxis (20, 21). Importantly, the perturbed mast cell maturation and anaphylaxis in *Pla2g3* ^–/–^ mice are recapitulated in mice lacking lipocalin-type PGD_2_ synthase (L-PGDS), which is expressed in stromal fibroblasts, or those lacking the PGD_2_ receptor DP1, which is induced in maturing mast cells. Thus, sPLA_2_-III secreted from mast cells hydrolyzes phospholipids in adjacent fibroblasts to release AA, which is then converted by fibroblastic L-PGDS to PGD_2_ that acts on DP1 on mast cells to promote their proper maturation (**Figure 5A**). This sPLA_2_-III-L-PGDS-DP1 paracrine circuit highlights a new aspect of PGD_2_-DP1 signaling in the regulation of mast cell maturation and thereby allergy and provides evidence for the long-standing proposal that sPLA_2_ acts as a paracrine coordinator of eicosanoid production in tissue microenvironments (20). Since EVs serve as a better hydrolytic target of sPLA_2_s (115, 116), it is also possible that sPLA_2_-III may act on mast cell-secreted EVs to release AA, which is incorporated into fibroblasts and utilized for L-PGDS-driven PGD_2_ generation.

Furthermore, mast cell-specific *Pla2g3*-deficient mice, as well as mast cell-deficient *Kit* ^W-sh^ mice reconstituted with mast cells prepared from global *Pla2g3^–^* ^/–^ mice, display a significant reduction of irritant contact dermatitis (ICD) and an aggravation of Th1-dependent CHS (21). The increased CHS response by sPLA_2_-III deficiency in mast cells depends, at least in part, on the reduced expression of H-PGDS and thereby reduced production of PGD_2_ due to immaturity of mast cells. During severe CHS responses, mast cells represent a source of IL-2 and IL-10, which amplify recruitment, maintenance, and function of T_reg_ cells that limit ear swelling and epidermal hyperplasia (117–120). H-PGDS deficiency in mast cells also abrogates the suppressive effect of mast cells on CHS, indicating that, in addition to the cytokines IL-2 and IL-10, the lipid mediator PGD_2_ serves as another negative regulator of the CHS responses. In support, mice lacking DP1 also display an exacerbation of CHS by affecting the expression of IL-10 in dendritic cells (60, 61). Taken together, sPLA_2_-III, which is secreted from mast cells, promotes mast cell maturation through the lipid-driven functional interaction with stromal fibroblasts, thereby facilitating acute anaphylactic and ICD reactions and limiting delayed CHS response.

### sPLA_2_-IIA Regulates Mast Cells Through Shaping of the Gut Microbiota

sPLA_2_-IIA (PLA2G2A) is a prototypic sPLA_2_ that is highly upregulated in various human tissues during inflammation such as rheumatoid arthritis, sepsis, and COVID-19 infection (121–123). While sPLA_2_-IIA promotes sterile inflammation by mobilizing pro-inflammatory lipid mediators or DAMPs (danger-associated molecular patterns) from EVs as an “inflammatory sPLA_2_” (115), it also efficiently degrades bacterial membranes (Gram-positive in particular), thereby playing a protective role against bacterial infection as a “bactericidal sPLA_2_” (124). In the context of mast cell biology, sPLA_2_-IIA was partially purified from rat mastocytoma RBL-2H3 cells (125), was detected immunohistochemically in secretory granules of rat serosal mast cells (126), triggered histamine release or PGD_2_ production by rat serosal mast cells when added exogenously at high concentrations (106, 107), and enhanced FcεRI-induced degranulation when overexpressed in RBL-2H3 cells (110, 111). However, as the *Pla2g2a* gene is naturally disrupted in C57BL/6 and 129 strains due to a natural frameshift mutation (127, 128), it had been difficult to evaluate the precise *in vivo* functions of endogenous sPLA_2_-IIA using a standard knockout strategy. Even in BALB/c mice, a strain that has an intact *Pla2g2a* gene (127), sPLA_2_-IIA expression is highly restricted to intestinal Paneth cells (129, 130). Although a trace level of *Pla2g2a* mRNA is induced in BALB/c-derived BMMCs after stimulation with SCF plus accessory cytokines *in vitro* (131), there has been no follow-up study showing that sPLA_2_-IIA is substantially expressed in mouse mast cells *in vivo*. This expression profile of sPLA_2_-IIA in BALB/c mice is in marked contrast to that in other animal species including humans and rats, in which sPLA_2_-IIA is expressed or induced in many tissues. Beyond these limitations, sPLA_2_-IIA-depleted (*Pla2g2a* ^–/–^) mice on the BALB/c background are best suited for analyzing the role of endogenous sPLA_2_-IIA in the intestine. Importantly, this new mouse model has unveiled a previously unrecognized, non-canonical action of intestinal sPLA_2_-IIA on mast cells *via* shaping of the gut microbiota.

Despite the restricted expression of sPLA_2_-IIA in the intestine, its genetic deletion unexpectedly leads to unusual changes in mast cell degranulation in distal skin. In a model of carcinogen-induced skin cancer, tumor development is markedly reduced, accompanied by a reduction of degranulated mast cells in the tumor tissue, in *Pla2g2a^–/–^* mice relative to *Pla2g2a* ^+/+^ mice (22). Surprisingly, cohousing of *Pla2g2a^–/–^* and *Pla2g2a* ^+/+^ mice in the same cages, which results in mixing of the microbiota between the genotypes through coprophagia, abolishes the skin cancer-related phenotypes. Of more interest, IgE/antigen-induced PCA is uniquely altered in *Pla2g2a^–/–^* mice depending on housing conditions; when *Pla2g2a* ^+/+^ and *Pla2g2a^–/–^* mice are housed in two different animal facilities, the PCA response in *Pla2g2a^–/–^* mice is reduced in one facility, while it is conversely elevated in the other facility, relative to *Pla2g2a* ^+/+^ mice (23). Furthermore, in both facilities, the opposite PCA phenotypes in *Pla2g2a^–/–^* mice are lost when they are cohoused with *Pla2g2a* ^+/+^ mice. Thus, the PCA phenotypes in *Pla2g2a^–/–^* mice are greatly influenced by housing conditions, implying that sPLA_2_-IIA, primary expressed in intestinal Paneth cells that secrete various antimicrobial peptides, contributes to shaping of the gut microbiota through its bactericidal activity, thereby secondarily affecting mast cell fate and associated allergic reaction in distal skin. Indeed, metagenome analysis of the stool revealed that several bacterial genera such as Gram-positive *Lachnospiraceae* and *Ruminococcaceae* and Gram-negative *Helicobacteraceae* and *Prevotellaceae* are differently distributed in *Pla2g2a* ^+/+^ and *Pla2g2a^–/–^* mice, and there is a better correlation between distinct PCA responses and *Ruminococcaceae* and *Mucispirillum* abundances in the two facilities. The alteration in gut microbiota in *Pla2g2a^–/–^* BALB/c mice, as well as in *PLA2G2A*-transgenic C57BL/6 mice in which human sPLA_2_-IIA is overexpressed systemically, also impact the severity of arthritis and psoriasis (22, 132). Thus, sPLA_2_-IIA acts as a host factor that is primarily expressed in the intestine and contributes to shaping of the gut microbiota, whose disturbance by *Pla2g2a* deletion or overexpression secondarily affects various diseases including those involving mast cells in proximal and distal tissues (**Figure 5B**). This concept opens a new avenue for the action modes of this classical sPLA_2_ and might be applicable to other sPLA_2_ isoforms expressed in the gastrointestinal tract or even in other anatomical sites such as the respiratory tract and skin.

Nonetheless, looking back the classical view that sPLA_2_-IIA is expressed in mast cells of rat and human, mast cell-autonomous roles of sPLA_2_-IIA should be reconsidered. Related to the observations that forcible overexpression or exogenous addition of sPLA_2_-IIA resulted in enhanced mast cell degranulation as described above (106–108, 110, 111), sPLA_2_ inhibitors suppressed degranulation by rat serosal mast cells (107) or LTC_4_ production by human lung mast cells (112), although the inhibitors used in those studies were not strictly specific for sPLA_2_-IIA. Alternatively, sPLA_2_-IIA released from activated mast cells, like that released from other cells such as platelets, leukocytes and epithelial cells, might contribute to propagation of inflammation by mobilizing lipid mediators from EVs (133), host defense against infection by degrading bacterial membranes (124), or regulation of cellular signaling by acting as a ligand for the sPLA_2_ receptor PLA2R1 (130, 134).

## Miscellaneous Roles of Other PLA_2_s in Mast Cell Biology

Several studies have demonstrated the potential roles of sPLA_2_-V (PLA2G5) and sPLA_2_-X (PLA2G10) in mast cells. Reportedly, sPLA_2_-V is released from antigen-activated BMMCs and then acts on neighboring fibroblasts to augment COX-1-dependent PGD_2_ biosynthesis (135–137), is localized to the perinuclear area in BMMCs (131), and augments FcεRI-induced PGD_2_ and LTC_4_ production when overexpressed in RBL-2H3 cells (110, 111). TLR2-induced, COX-2-dependent delayed PGD_2_ generation is partially reduced in BMMCs from sPLA_2_-V-deficient (*Pla2g5* ^–/–^) mice, where sPLA_2_-V may act in cooperation with cPLA_2_α (138). In addition, sPLA_2_-X is detected in BMMCs, and IL-13 induction in response to IL-33 is substantially impaired in BMMCs from sPLA_2_-X-deficient (*Pla2g10* ^–/–^) mice (139). Importantly, *Pla2g5* ^–/–^ and *Pla2g10* ^–/–^ mice are both protected from airway inflammation induced by allergen challenge through the mechanisms involving ILC2 cells, M2 macrophages, eosinophils, airway epithelial cells, and possibly mast cells (139–145). However, other studies showed that the expression levels of sPLA_2_-V and -X in BMMCs are rather low and that FcεRI-induced BMMC activation *ex vivo* and PCA reaction *in vivo* are not significantly affected in *Pla2g5* ^–/–^ and *Pla2g10* ^–/–^ mice (17, 20, 146). To reconcile these inconsistencies, it is necessary to clarify whether these two sPLA_2_s are expressed in a certain population of mast cells and play specific roles therein under particular pathophysiological conditions.

Several lines of evidence argue the involvement of mast cells in cardiometabolic diseases (147). Since activated mast cells produce PGD_2_ and LTB_4_, which have been implicated in cardiovascular pathology including atherosclerosis (148, 149), it is likely that cPLA_2_α contributes to the disease by supplying these eicosanoids in atherosclerotic plaques. Indeed, global *Pla2g4a* ^–/–^ mice are protected from the development of atherosclerosis (150). However, the contribution of cPLA_2_α expressed in mast cells to the disease remains unknown and should be confirmed using mast cell-specific *Pla2g4a* ^–/–^ mice. Modified or oxidized low-density lipoprotein (LDL) has been shown to induce mast cell activation, resulting in cytokine secretion and subsequent leucocyte recruitment, presumably through TLR4 (151). As a component of modified LDL, the lysophospholipid mediator LPA can induce mast cell activation, resulting in the release of tryptase and chemokines (152). Many LPA species could be detected in the atherosclerotic lesion, where mast cells also reside (152, 153). Since several sPLA_2_s have the capacity to generate modified LDL with a pro-atherogenic potential *in vitro* (154–157), it is tempting to speculate that LPA produced in LDL by these sPLA_2_s may contribute to the development of atherosclerosis through activating aortic mast cells. Although several studies using mice overexpressing sPLA_2_-IIA or those lacking sPLA_2_-V or sPLA_2_-X have proposed the potential roles of these sPLA_2_s in atherosclerosis, none of them has provided evidence that these sPLA_2_s generate modified, pro-atherogenic LDL *in vivo* (158–162). Therefore, it is still unclear whether sPLA_2_s could promote atherosclerosis development by modifying LDL and generating LPA, and if so, which sPLA_2_ isoform(s) would be truly responsible for this event in the context of mast cell activation.

Group IVD cPLA_2_ (cPLA_2_δ or PLA2G4D) was initially identified as a keratinocyte-specific cPLA_2_ isoform that is highly induced during psoriasis (163). It has recently been shown that cPLA_2_δ is expressed in mast cells of psoriatic patients and is released extracellularly *via* mast cell-derived EVs to be transferred into adjacent Langerhans cells (164). The EV-driven cPLA_2_δ captured by Langerhans cells generates neolipid antigens, which are then presented on CD1a to activate lipid-specific CD1a-reactive T cells, leading to induction of the Th17 cytokines IL-17A and IL-22. Although these results have provided a model whereby cPLA_2_δ promotes psoriasis pathology, it remains unclear which lipid metabolites produced by cPLA_2_δ can act as neolipid antigens. Since cPLA_2_δ exhibits PLA_1_ activity in preference to PLA_2_ activity (165), certain lipid metabolites generated by the PLA_1_ reaction might underlie the function of cPLA_2_δ. Further, given that cPLA_2_δ is expressed in epidermal keratinocytes rather than in mast cells and that CD1a is present in humans but not in mice, the regulatory roles of cPLA_2_δ in psoriasis or other skin diseases such as atopic dermatitis in the context of mast cells need further exploration.

Mast cells also express group VIA Ca^2+^-independent PLA_2_ (iPLA_2_β, also known as PLA2G6 or PNPLA9). Pharmacologic inhibition of iPLA_2_β by bromoenol lactone (BEL), a well-known iPLA_2_ inhibitor, attenuates IgE/antigen-stimulated mast cell exocytosis (166). However, mice deficient in iPLA_2_β (*Pla2g6* ^-/-^) show a normal PCA response *in vivo*, and *Pla2g6* ^-/-^ BMMCs exhibit normal degranulation, eicosanoid generation, and cytokine expression *in vitro* (17), arguing against the contribution of iPLA_2_β to mast cell development and functions. Thus, caution should be exercised when interpreting the results obtained from studies using BEL or other iPLA_2_ inhibitors. Alternatively, considering that the iPLA_2_ family includes nine isoforms, some BEL-sensitive iPLA_2_ isoform(s) other than iPLA_2_β, such as group VIB iPLA_2_γ (PNPLA8) which is expressed in BMMCs more abundantly than iPLA_2_β (18), might be involved in the regulation of mast cells.

Interestingly, silencing of adipose triglyceride lipase (ATGL, also referred to as PNPLA2 or iPLA_2_ζ), a member of the iPLA_2_ family that plays an essential role in lipolysis and thereby energy metabolism by hydrolyzing triglycerides stored in lipid droplets (167), results in the reduction of PGD_2_ and LTC_4_ synthesis more efficiently than silencing of cPLA_2_α in human mast cells derived from blood CD34^+^ progenitors (168). These results suggest that the AA released from triglycerides by ATGL is coupled with eicosanoid synthesis in mast cells, underscoring a functional link between energy homeostasis and eicosanoid signaling. However, given the essential role of cPLA_2_α in eicosanoid generation in mast cells as revealed by studies using *Pla2g4a* ^-/-^ mice (see above), the following possibilities should also be taken into consideration: (i) mouse and human mast cells might have distinct dependence on cPLA_2_α and ATGL; (ii) the AA released by ATGL from lipid droplets might be firstly incorporated into membrane phospholipids by lysophospholipid acyltransferases and then cleaved out by cPLA_2_α toward eicosanoid synthesis; or (iii) ATGL-mediated energy supply might be required for appropriate cPLA_2_α-driven eicosanoid generation. Also, it is important to clarify whether ATGL in mast cells is involved in the regulation of eicosanoid synthesis or other effector functions under certain *in vivo* conditions.

## Concluding Remarks

In this article, we have highlighted current understanding of the pathophysiological roles of several PLA_2_s and associated bioactive lipids in mast cell biology, focusing mainly on the findings obtained from studies using gene-manipulated mice in combination with comprehensive lipidomics. As a future prospect, it is important to translate these findings obtained from experimental animals into humans. It is possible that the functions of mast cells may also be affected by other PLA_2_s or bioactive lipids not described in this article, by bioenergetics coupled with lipogenesis, lipolysis and fatty acid β-oxidation, and by lipid composition in membrane microdomains that could affect signal potency through FcϵRI or other receptors. A full understanding of lipid networks in relation to mast cells, allergy, and other mast cell-dependent biological events should be further elucidated using advanced techniques such as spatiotemporal lipid imaging, untargeted lipidomics, and novel pharmacological tools to manipulate the activity or expression of particular PLA_2_ subtypes that would have a potential to sequester allergic or other diseases. Further research will lead to a better understanding of the overall picture of the regulation of mast cells by lipids, hopefully allowing the prophylactic and/or therapeutic application of novel agents that target specific PLA_2_-driven lipid pathways to human diseases.

## Author Contributions

MM and YT wrote the manuscript and prepared Figures. All authors have read and agreed to the published version of the manuscript.

## Funding

This work was supported by Grants-in-Aid for Scientific Research JP19K22483 and JP20H05691 (to MM) and JP18K06624 (to YT) from Japan Society for the Promotion of Science, and AMED-CREST JP21gm1210013 from the Japan Agency for Medical Research and Development (to MM).

## Conflict of Interest

The authors declare that the research was conducted in the absence of any commercial and financial relationships that could be construed as a potential conflict of interest.

## Publisher’s Note

All claims expressed in this article are solely those of the authors and do not necessarily represent those of their affiliated organizations, or those of the publisher, the editors and the reviewers. Any product that may be evaluated in this article, or claim that may be made by its manufacturer, is not guaranteed or endorsed by the publisher.
